# Discrepancy of the law on disaster emergency in Indonesia: In search of an integrated law

**DOI:** 10.4102/jamba.v16i1.1437

**Published:** 2024-02-01

**Authors:** M. Yakub Aiyub Kadir, Teuku Ahmad Dadek, Azhari Yahya, Aditya Rivaldi

**Affiliations:** 1 Department of International Law, Faculty of Law, Syiah Kuala University, Banda Aceh, Indonesia; 2 Aceh Regional Development Planning Body, Banda Aceh, Indonesia; 3 Department of Civil Law, Faculty of Law, Syiah Kuala University, Banda Aceh, Indonesia

**Keywords:** COVID-19, management, disaster emergency, law, Indonesia

## Abstract

The emergency status is a critical factor in handling disasters in Indonesia, particularly for coronavirus disease 2019 (COVID-19). It provides adequate access for the Government to reduce legal bureaucracy and facilitate expenditure. However, it has faced difficulties in its application as it does not have an explicit reference to existing emergency-related disaster laws. This article investigates this discrepancy in terms of the meaning of emergency status within the legal framework in Indonesia. This study uses the doctrinal method to explore six various laws related to disaster emergencies and analyse their discrepancies that have adversely impacted the management of COVID-19. The article finds that the term ‘disaster emergencies’ in the Indonesian legal system has different legal interpretations and a lack of standardisation, making their execution difficult.

**Contribution:**

This article significantly highlights the normative issues of determining disaster emergencies and their status in six different disaster-related laws. It provides an alternative approach to mainstream thinking by proposing amendments to the Infectious Disease Outbreak Law as an integrated law to ensure legal certainty, benefit, and fairness for the people in handling potential pandemics in Indonesia in the years to come.

## Introduction

Since coronavirus disease 2019 (COVID-19) emerged in China and was declared a pandemic by the World Health Organization (WHO 2020) on 11 March 2020, 228 countries have been affected, resulting in 5 821 004 deaths and 412 351 279 infections, while in Indonesia 4 966 046 persons were infected and 145 622 died in 365 districts or cities (Covid19.go.id 2021). This situation continues to persist without anyone knowing when it will come to a halt. The Government of Indonesia’s primary response to COVID-19 has been to convert the natural hazard into a legal event with a process for evaluating the status of a disaster emergency (*The Disaster Management Act*, ss. 1–18).

The government determined the COVID-19 disaster’s emergency status using Law No. 6 of 2018 on Health Quarantine (UUKK), an article regulating Public Health Emergencies, and Law No. 24 of 2007 on Disaster Management contains the legal notion of Disaster Emergency Status (Asikin, 2012).

Additionally, Indonesia already has Law No. 4 of 1984 on Infectious Disease Outbreaks (WPM Law), which should serve as the primary legal framework for dealing with the COVID-19 outbreak. However, this law is inadequate. Since 2012, the law has been academically drafted for alterations or revisions for various reasons. This law would serve as a foundation for enhancing future management and ensuring legal sufficiency.

The national disaster management law no 24/2007 categorises disaster management into three stages: (1) pre-disaster, (2) emergency response, and (3) post-disaster. These stages do not indicate a dichotomy between them, but rather show that they are interrelated. What is critical is that this level requires delegation to the government to take swift action in a disaster emergency. This stage is a normal cycle in the national disaster management system (Law no 24/2007). While there are numerous implementation variants around the international globe, what is critical is that disaster and its management are a connected series, not a series of isolated incidents (Janpatar 2010; Rahardjo 2006).

The stages will employ uncommon or conventional legislation compared to disaster management during the pre-and post-disaster phases. In an emergency system, each declaration of an emergency allows the government to disregard several fundamental principles, including restrictions on the Constitution’s articles of independence and freedom, suspension of human rights, and violations of the law governing the procurement of goods and services for crisis management of an emergency nature. This article explores the concept and regulation of Indonesia’s Disaster Emergency Law and its implementation throughout the COVID-19 period by examining six disaster-related laws in Indonesia.

## Method

The approach utilised is doctrinal, involving the evaluation of discrepancy among the norms of six disaster-related laws. The differences in the concept of disaster emergency law in the six disaster-related laws can be seen in Table 1. These laws encompass legal principles, standards, statutory rules, court decisions, agreements, and doctrines (teachings) (Soekanto & Mamudji 2001). Furthermore, the historical aspect of imposing an emergency status in Indonesia is also examined to gain a comprehensive understanding of the concept. This understanding, in turn, contributes to varying interpretations of emergency status and its relevance to the management of the COVID-19 pandemic (Marzuki 2017).

**TABLE 1:** Differences in the concept of disaster emergency law in the six disaster-related laws.

No	Regulation	Centralised groups						Decentralised groups					
		Law no. 4 of 1984 about outbreak of transmitted desease		Law no. 36 of 2009 concerning health		Law No. 6 of 2018 concerning health quarantine		Law no. 24 of 2007 concerning disaster management		Law no. 32 of 2004 concerning regional administration		Government regulation no. 1 of 2020	
5	Term of emergency	1	Plague area	1	Outbreak, eruption or extraordinary events	1	Public health emergency	1	Emergency response	1	Certain circumstances, emergencies, extraordinary circumstances	1	Emergency
6	Emergency management	1	Health	1	Emergency nutritional adequacy	1	Determination of disease	1	Assess and determine the emergency	1	Drafting regulations outside the regional legislation program	1	Refocusing
		2	Compensation	2	Directing health facilities for handling emergency response	2	Quarantine, isolation, administering vaccines, referrals, disinfection and decontamination of people according to indications	2	Rescue, evacuation, fulfillment of basic needs, protection of vulnerable groups, recovery	2	State budget can provide emergency funds to help regions	2	State income including tax policy
		3	Medical award	3	Disaster emergency response	3	Large-scale social restrictions	3	Deployment of human resources	3	Home provision and rehabilitation	3	Expenditure including regional finance
		4	Reporting	4	Provide medicine	4	Disinfection, decontamination, disinfection and/or derealisation of transportation means and goods	4	Deployment of equipment, logistics deployment	4	Basic needs and trauma recovery	4	Financing
		5	Criminal	5	Medical immunity	5	Health, security, and control of environmental media	5	Immigration, excise, and quarantine, rescue and command to command sectors/agencies	5	Community empowerment	5	Handling anticipation of the national economy
				6	Medical emergency	6	Medical services, food, and others during quarantine	6	Licensing; procurement of goods/services, management and accountability of money and/or goods	6	Archive rescue, agriculture	6	Financial institutions

*Source:* Dadek, T.A., Jalil, H., Syahbandir, M. & Kadir, M.Y.A., 2023, *Politics of Law against Covid-19 in Indonesia*, Syiah Kuala University, Banda Aceh

## Results and discussion

### Defining of disaster emergency

The Law on Disaster Emergencies originated from and was incorporated into *staatsnoodrechs* or countries in a state of emergency or danger, but through its development, most notably following the enactment of the Undang Undang tentang Penganngulangan bencana or Law on Disaster Manajement (UUPB) Law Number 24 of 2007 concerning disasters, the stipulation of disaster emergencies has developed its legal conception and law in a medical emergency (ed. Sihombing 1996) (See Table 2).

**TABLE 2:** Implementing government regulations based on UUPB.

No	Article and section	Type of regulation	Note
**A.**	**Law of the Republic of Indonesia number 24 of 2007 concerning disaster management (UUPB)**		
1.	Article 30 paragraph 3: Further provisions concerning the implementation of disaster management activities by international institutions and foreign non-governmental organisations shall be governed by a Government Regulation.	Government Regulation of the Republic of Indonesia Number 23 of 2008 concerning the Participation of International Institutions and Foreign Non-Governmental Organisations in Disaster Management	Already published
2.	Article 50 paragraph 2: Further provisions concerning easy access as referred to in paragraph (1) shall be governed by a Government Regulation	Government regulation	Not yet published
3.	Article 69 paragraph 4: The Procedures and amount of aid as referred to in paragraph (1) and paragraph (2) shall be regulated further by a Government Regulation.	Government regulation	Not yet published
4.	Article 50 paragraph (2), Article 58 paragraph (2), and Article 59 paragraph (2): need to stipulate a Government Regulation concerning the Implementation of Disaster Management.	Government Regulation of the Republic of Indonesia No. 21 of 2008 on implementation of disaster control.	Already published
5.	Article 63: Further provisions concerning disaster management funds handling mechanism as referred to in Article 60 to Article 62 shall be governed by a Government Regulation.	Government Regulation of the Republic of Indonesia No. 22/2008 concerning disaster aid financing and management.	Already published
6.	Article 7 paragraph 3: Further provisions concerning decision on disaster status and level as referred to in paragraph (2) shall be stipulated by a Presidential Regulation	Presidential Decree	Not yet published
7.	Article 17: Further provisions concerning the establishment, functions, tasks, organisational structure, and working arrangement of National Disaster Management Agency shall be stipulated by a Presidential Regulation.	Regulation of President of Republic of Indonesia no. 8 of 2008 concerning national disaster management agency which was later amended by Regulation of President of Republic of Indonesia Number 1 of 2019 concerning National Disaster Management Agency	Already published

*Source:* Dadek, T.A., Jalil, H., Syahbandir, M. & Kadir, M.Y.A., 2023, *Politics of Law against Covid-19 in Indonesia*, Syiah Kuala University, Banda Aceh

Since 1945, the Indonesian legal system has used three distinct phrases to refer to emergencies: a state of danger, a compelling exigency, and a state of emergency. Asshiddiqie (2007:97) argued that the premise or principle of declaring a state of emergency is a constitutional prerogative granted to the state to limit human rights to expedite recovery and impose temporary limits to overcome catastrophic problems and restore normal conditions. Because the constitution vests this enormous authority in the legislature, the law also establishes limitations or conditions on when that authority may be employed, such as a time limit or the stipulation of circumstances during a situation of danger or emergency. The regulation’s objective is to balance unbridled authority and quantifiable constraints, following the principle of balance legislation (*evenwichts* theory) (Asshiddiqie 2007).

According to the Theory of the Law of Equilibrium, danger conditions are abnormal conditions; therefore, legal remedies must also be abnormal and extraordinary; in normal circumstances, the authorities’ actions are illegal or against the law; however, when abnormal conditions return to normal, the rulers’ actions become valid and justifiable (ed. Sihombing 1996:2). The appropriate criteria for defining the emergency must be detailed not only in the Constitution but also in special regulations. The Indonesian Constitutional Council has established what may be uttered during a state of emergency (Simamora 2010:58–70).

An emergency is a period of chaos that must be regulated normally to avoid multiple legal interpretations in dealing with the situation; consequently, it is necessary to regulate the mechanisms and procedures that allow the state to function according to the wishes of the state as compiled during regular times and the Constitution. An emergency has the potential to give rise to unconstitutional political interpretations, multiple interpretations to define a situation that is easily classified as an emergency, and the potential to give birth to a dictatorship. Sembiring has attempted to formulate several requirements, including the following: firstly, the existence of the State’s highest interest, namely the State’s highest interest, for the State’s existence, which is jeopardised in an emergency (*het hoogste staatsbelang – het bestaan zelf van de staat – stond on het spel en was afhankelijk van het al of niet maken der getroffen regeling*). Secondly, it is critical to enact emergency measures to balance out aberrant conditions (*deze regeling noodzakelijk was*). Thirdly, the Emergency Law’s validity is transitory (*provosoir*), or it must be swiftly restored to normal conditions, but the temporary term is not specified. Fourthly, the Parliament cannot convene in the event of an emergency but must participate at the appropriate time (ed. Sihombing 1996).

Asshiddiqie and Safa’at (2006) use the term *staatsnoodrecht* to refer to a country’s legal system during a state of emergency. They argue that many *statemakers* and legal systems consider the state to be in a normal state, and thus many laws are drafted in normal circumstances, even if the state may be in an abnormal state. An emergency is also quite possible; this abnormality or emergency might be triggered by political situations both within and outside the country, social conditions such as conflict and civil war, or natural calamities. Since 1945, the Indonesian legal system has used three distinct phrases to refer to emergencies: a state of danger, a compelling exigency, and a state of emergency (see Table 3).

**TABLE 3:** Comparison of hazards, emergencies and disaster emergency status.

No	Danger situation	State emergency	Disaster emergency
1	Compelling urgency (force majeure)	Non-disaster and disaster	Disaster
2	The authority regulates in the 1945 Constitution	The authority regulates in the 1945 Constitution and government regulations	The authority regulates in Law No 24 of 2007 on Disaster Management
3	President’s authority	President’s Authority	Authority of the President, Regional head based on the government level.
4	Political based	Political and security, and disaster	Disaster
5	Centralisation	Centralisation	Centralisation and autonomy

*Source:* Dadek, T.A., Jalil, H., Syahbandir, M. & Kadir, M.Y.A., 2023, *Politics of Law against Covid-19 in Indonesia*, Syiah Kuala University, Banda Aceh

According to Article 22 of the 1945 Constitution:

[*I*]n the event of a compelling emergency, the President has the authority to prescribe government regulations in lieu of law and must gain agreement from the House of Representatives in the subsequent trial.

The Republic of United States of Indonesia’s (RIS) Constitution also governs emergencies and hazards; however, the RIS Constitution defines them as ‘a state of war (article 127), an urgent emergency (article 96), and a condition of danger (article 129)’ (DPRRI 1950:1–47).

The three systems of the decision have various legal processes in ‘a condition of danger’ and ‘emergency’, which will be fully under the power of the President as head of State per the spirit of centralisation and unification. Meanwhile, ‘state of emergency and disaster level’ or ‘status of disaster emergency’ becomes a tiered authority following the spirit of decentralisation and autonomy.

### Situations of danger and emergencies

Article 12 of the 1945 Constitution states that ‘The President declares a state of danger. The circumstances and effects of a state danger are regulated by legislation’. The law that has been utilised to define a state of danger thus far is the Law Number 74 of 1957 on the repeal of ‘*Regeling Po De Staat Van Oorlog En Beleg*’ and the enactment of ‘State of Danger’.

Article 1 paragraph (1) states that the President, on the recommendation of the Council of Ministers, may declare the entire territory or a portion of the territory of Indonesia in a state of danger with a state of emergency or a state of war if security or law and order in the entire territory or a portion of the territory of Indonesia are threatened by rebellions, riots, or natural hazards that are feared to be insurmountable by tools.

Ihsanuddin (2020: 1–9) stipulates that the government does not use the term ‘state of danger’ concerning COVID-19, even though the government has contemplated using this legal tool for short-term COVID-19 prevention. There is a reason why experts believe that using the term ‘civil emergency’ to prevent COVID-19 is inappropriate for two reasons: firstly, it conjures up images of authoritarian regimes, and secondly, it avoids state legal responsibilities because the problem of restricting people’s movements can be addressed through *Act No. 3 of 2018* with regards to quarantine (Dzulfikar 2020:19–22).

### A compelling exigency

The constitution makes no express provision for disasters, much fewer epidemics. The Indonesian Constitution provides only for a ‘compelling exigency’ process, which according to article 22 of Indonesian Constitution 1945 allows the President to issue a Government Regulation in Lieu of Law (PERPPU) and must receive approval from the Dewan Perwakilan Rakyat or The House of Representatives of the Republic of Indonesia (DPR). However, if there is no such approval, these government regulations shall be revoked. The 1945 Constitution does not specify what constitutes a coercive urgency that endangers the state.

The Republic of Indonesia Constitutional Court’s Decision number 138/PUU-VII/2009 establishes three elements for compelling urgency, namely:

1. The existence of urgent circumstances impairing or endangering national interests.
2. There is a legal void or insufficient legal protection.
3. The DPR’s implementation of the law-making process through the legislative mechanism.

This has been demonstrated by the Government’s response to the COVID-19 outbreak, namely the issuing of Government Regulation in Lieu of Law of the Republic of Indonesia Number 1 of 2020 concerning State Financial Policy and Financial System Stability for Handling the COVID-19 pandemic and/or in facing threats that endanger the economy, which focuses on three areas: (1) handling COVID-19, (2) the state financial system and economy, and (3) state financial stability (Effendi 2020:67–79).

### The development of disaster emergency law in Indonesia

Indonesia’s Disaster Emergency Law was enacted following the passage of Law No. 24 of 2007 on Disaster Management (UUPB). The UUPB was founded and heavily affected by the 26 December 2004 earthquake and tsunami in Aceh. Prior to the UUPB, disaster management in Indonesia lacked a strong legal foundation; disaster management was considered a minor component of the health, environment, and other systems (Dadek, Rinaldi & Sulaiman 2020:13).

At the time of independence, various laws managing crises or risks existed, which influenced the Law on disaster emergency development. Numerous regulations include the Law No. 6 of 1946 on Dangerous Conditions, Law of the Republic of Indonesia No. 74 of 1957 (74/1957) on the repeal of the ‘*Regling Po De Staat Van Oorlog En Beleg*’ and the Determination of Dangerous Conditions, and Government regulation No. 23 of 1959.

### Disaster and health emergency designation

Dadek et al. (2021:23–42) argued that since independence, the Government has issued several decrees regarding the status of a disaster emergency. The issuance of the disaster emergency is intended as a legal basis that the Government takes over the disaster management, not as a legal basis to be regulated in stages and to provide legal breadth and authority to facilitate the handling of the emergency. Since the issuance of the UUPB, the concept of disaster emergency law has become clear and has legal certainty, but until now, it is still constrained by the mechanism and its benchmarks. After independence, the Government of Indonesia issued several legal regulations regarding the determination of a national disaster emergency status, including: Presidential Decree No. 254 of 1966 concerning the Annual Disaster Eruption of Mount Awu in Sangir Talaud Regency (North Sulawesi), Presidential Decree in response to the Aceh Earthquake and Tsunami, Presidential Decree No. 371 of 1961 declaring the eruption of Mount Merapi, Presidential Decree No. 1992 on the Determination of Natural Hazards in Flores, and Presidential Decree No. 12 of 2020 concerning the Determination of Non-Natural Hazards for the Spread of COVID-19 as a National Disaster.

### Diverse concepts of disaster emergency in Indonesia

The concept of Law (*genuine legal concepts*) is defined as ‘a constructive and systematic concept used to understand a Rule of Law (e.g. the concepts of rights, obligations, legal relations, legal institutions, engagements, marriages, inheritance and buying, and selling)’ (Wignjosoebroto 1974:89–98).

### Outbreak area

Although the WPM Law utilises the phrase ‘plague area’, there is no legal definition of what constitutes an ‘outbreak area’. The legal definition of an outbreak area is contained in the Republic of Indonesia’s Government Regulation No. 40 of 1991 on the Control of Infectious Disease Outbreaks, which defines an outbreak area as ‘a region deemed to be infected with an epidemic’. Additionally, this government regulation identifies the legal definition of an Kejadian Luar Biasa or extraordinary events (KLB), which is:

[*T*]he emergence or increase in the incidence of illness/death that is epidemiologically significant in a particular area during a specified time period, and is a situation that can result in an outbreak. (Indonesian Government 1991:s.1)

Although very few criminal acts are committed, the Wabah Penyakit Menular or Infectious Disease Outbreaks (WPM) Law No. 4 of 1984 on Infectious Disease Outbreaks provides that:

Anyone who intentionally obstructs the implementation of epidemic control will face a maximum sentence of one year in prison and/or a maximum fine of Rp. 1 000 000 (one million rupiah).

Then:

Anyone who, by negligence, obstructs the implementation of epidemic control is subject to a maximum sentence of six (six) months in prison and/or a maximum fine of Rp. 500 000 (five hundred thousand rupiahs).

The primary issue is that there is no specific legal definition of wilful obstruction; WPM Law has already defined what constitutes a crime and a violation based on the perpetrator’s intent, whether intentional or just indulgent.

### Emergencies, outbreaks, eruptions, or extraordinary events (KLB) based on laws of health

Law of the Republic of Indonesia Number 36 Year 2009 concerning health uses three legal terms: Emergency, Outbreak, Eruption, or Extraordinary Events. Numerous chapters explain the concept of a state of emergency but are deficient in terms of the rule requiring health facilities to prioritise emergency patients (threatening) life or disability, the prohibition of refusing patients, as well as the obligation of the government to provide drugs and a source power, and the facilities’ time of emergency until post-emergency. The health care professionals also do not have the right to sue for negligence during a state of emergency (Prasetio 2021).

Additionally, the UUK controls efforts to prevent and respond to outbreaks, particularly their health or medical consequences. The government has the authority under Article 36, paragraph 1 to ‘declare a region to be in a state of the epidemic, eruption, or unusual occurrence (KLB)’. The UUK only regulates what the Government, public sector, and private sector must do in an emergency and post-disaster situation; it does not regulate who declares an emergency, how the mechanism is implemented, or what facilities can be obtained to expedite the situation, except for the procurement of medications on the basis of legal concepts.

### Public health emergency

The Republic of Indonesia’s Public Health Emergency Law, No. 6 of 2018 on Health Quarantine defines a Public Health Emergency as:

[*A*]n extraordinary event marked by the spread of infectious diseases and/or events caused by nuclear radiation, biological pollution, chemical contamination, bioterrorism, or food that poses a health hazard and has the potential to spread across regions or countries.

This draft is used as the basis for executing quarantine and sealing the country’s borders, and it is the Central Government’s authority. This requires that the sort of disease that will cause a public health emergency be determined and that this determination takes into account the social and economic consequences. Indeed, Public Health Emergencies have a high association with regional quarantine and Pembatasan Sosial Berskala Besar or Large- Scale Social Restrictions (PSBB) and regulated criminal sanctions.

### Legal concepts in emergencies according to UUPB

The Law of the Republic of Indonesia No. 24 of 2007 on Disaster Management (UUPB), is currently being discussed for revision for a variety of reasons. The administration has drafted an academic paper but has not yet placed it in the National Prolega (DPRRI 2019). The UUPB has clearly defined the stages of a disaster, including pre-disaster, disaster emergency, and post-disaster, as well as the necessary activities. Although the UUPB regulates the legal system for assessing disaster emergencies, the UUPB remains vulnerable to physical disasters.

The UUPB’s notion of disaster emergency law is as follows:

1. Disaster emergency response can be defined as an immediate or direct response to a disaster that has occurred and is characterised by physical handling actions.
2. Disaster Emergency Assistance is defined as ‘an endeavor to address fundamental requirements during times of disaster’.
3. A disaster emergency is ‘a state determined by the government for a specified amount of time-based on the advice of the agency charged with disaster response’.

The definition of disaster emergency status remains geared around disaster management institutions, in this case, Badan National Penanggulangan Bencana or National Agency for Disaster Management (BNPB) or Badan Penanggulangan Bencana Daerah or Regional Agency for Disaster Management (BPBD). The activities include the following: (1) rapid and precise assessment of the location, the extent of damage, and available resources; (2) determination of the emergency status of a disaster; (3) rescue and evacuation of disaster-affected communities; (4) provision of basic necessities; (5) protection of vulnerable groups; and (6) immediate recovery of vital infrastructure and facilities (DPRRI 2019).

What are the legal advantages of declaring a disaster emergency? According to UUPB, BNPB or BPBD has easy access to the following capabilities: (1) mobilisation of human resources; (2) equipment deployment; (3) logistics deployment; (4) immigration, excise, and quarantine; (5) licensing; (6) procurement of goods or services; (7) management and accountability of money and/or goods; (8) rescue; and (9) command to command the sector or institution.

While the UUPB’s concept of Disaster Emergency is adequate, it does not provide legal certainty for dealing with epidemic disasters, particularly COVID-19, because it is still too focused on physical disasters, particularly emergency response activities, and thus cannot be used to implement law-enforcement processes or health protocols, particularly quarantine activities.

### Emergency concept under the Government Regulation in Lieu of Law No. 1 of 2020

Government Regulation in lieu of Law of The Republic of Indonesia No. 1 of 2020 Concerning State Financial Policy and Financial System Stability to control the COVID-19 Pandemic and/or in Response to Dangerous Threats to The National Economy and/or the Stability of the Financial System was issued with reasons that already met the criteria of ‘compelling exigency’ that is:

1. The spread of COVID-19 has become a pandemic and has entered Indonesia and has had a very large impact; that the implications of the COVID-19 pandemic are a slowdown in national economic growth.
2. It has led to a decline in the economy, state revenues, and increased spending, especially in handling health and the national economy.
3. Deteriorating state financial system.

This diversity cannot be separated from the legal politics of the Indonesian Government, which has not been systematical, integrated, and based on the ability to formulate laws intellectually. Syahriza Alkohir Anggoro quoted Natsir as saying that *rechtpolitiek* is ‘a political action of the Government through law based on the principles of the Rule of Law and the principle of democracy’ (Anggoro 2019:77–86).

### The UUPD’s concept of disaster emergency law

The following matters are addressed in Law No. 23 of 2014 on disaster and disaster emergencies:

1. Emergency funds are those that may be included in the Anggaran Pendapatan dan Belanja Negara or Indonesian State Budget (APBN) but are only distributed following a calamity. Emergency funding can also be included in the APBD, and disasters are a common basis for APBD adjustments.
2. The UUPD defines ‘emergency situation’ in its explication of Article 316, which establishes the conditions for an ‘emergency situation’ that fits at least the following criteria: a) it is not a routine activity of the regional government and cannot be forecasted in advance; b) It is not expected to occur regularly; c) It is beyond the regional government’s control and influence; and d) it has a major impact on the budget for emergency recovery.

### Disaster emergency and legal certainty

Certainty is an inherent characteristic of law, particularly positive and written legal norms. Without the value of certainty, law loses its meaning because it can no longer be used to govern everyone’s behaviour (Wantu 2007:19). Certainty is referred to as one of the objectives of law. Legal certainty is described as the clarity of standards to the point where they can be utilised as a guide for those who are regulated (Leawoods 2000:489–515).

**FIGURE 1:**
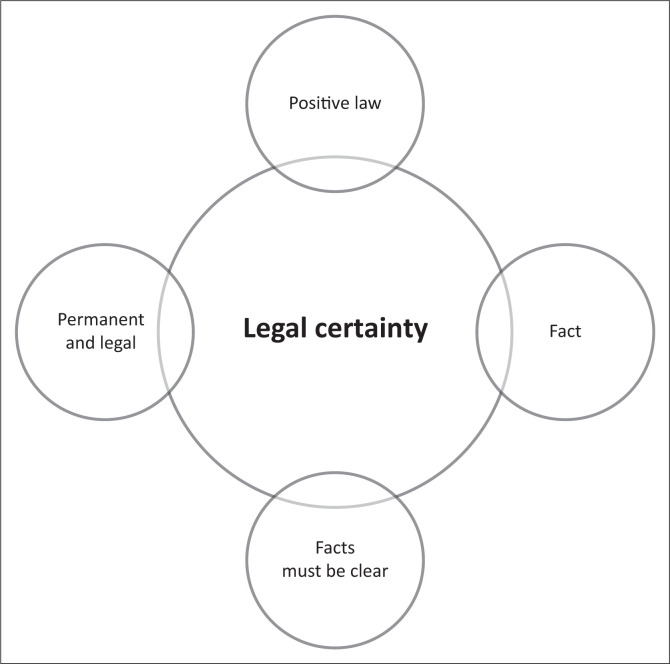
The four elements of legal certainty by Gustav Radbruch.

Gustav Leawoods (2000:489–515), a well-regarded intellectual person in the field of legal certainty, stated that legal certainty must satisfy four critical and fundamental requirements connected to the definition of legal certainty (see Figure 1 and Table 4):

1. The law is positive, meaning it is written.
2. Laws are factual in the sense that they are founded on reality.
3. Facts must be succinctly stated, avoiding ambiguity in interpretation, and simple to apply and implement.
4. Positive law is impervious to change, implying that it must be fixed in the mechanism of formation and the authorised institution with the authority to modify.

**TABLE 4:** Technical characteristics of legal certainty.

No	Characteristics of legal certainty	Meaning
1	Positive law	Law that has been enacted by a duly authorised legislature.
2	Fact	An event that actually happened, or a statement presented as objective truth.
3	Facts must be clear	Avoid misinterpretation; easy to implement
4	Permanent and legal	Everlasting, especially without significant change and established by or founded upon law; lawful

*Source:* Dadek, T.A., Jalil, H., Syahbandir, M. & Kadir, M.Y.A., 2023, *Politics of Law against Covid-19 in Indonesia*, Syiah Kuala University, Banda Aceh

Legal certainty is greatly impacted by positivism’s flow, in which the validity of legislation is determined by objective legal principles that are fully divorced from morality. A law is deemed invalid if it fails to satisfy these standards of pure law.

Fuller (1971:76) established eight criteria that a law must meet; if they are not met, the law ceases to be a law; in other words, there must be legal certainty. The following are the eight principles:

1. A legal system composed of regulations that are not reliant on erroneous judgments in certain instances.
2. The regulation is made public.
3. Not retroactive, as this would jeopardise the system’s integrity.
4. Formulated in a way that the general public understands.
5. There should be no regulations that contradict one another.
6. Must not demand more than is feasible.
7. Should not be regularly altered.
8. Regulations must be consistent with their day-to-day implementation.

There must be consistency between rules and their execution. Legal certainty ensures that the law is correctly applied and requires legal arrangements in legislation to be formed by competent and authorised legal parties to ensure that they have a legal basis and are genuinely obeyed and operate (Zainal 2012:45).

Legal certainty is defined in Utrecht in two ways: (1) the existence of general legal rules that inform individuals about what actions may or may not be taken, and (2) legal security for individuals against government arbitrariness because the existence of general rules informs individuals about what the state may charge or do to them (Syahrani 1999:23). Rahardjo (2006:134) stated that while legal certainty has developed into a form of philosophy in legal life, other processes, notably psychological and political processes, are required for legal certainty to exist. Socio-historically, the issue of legal certainty arose along with the capitalist economy’s production structure. Legal certainty is essentially a law that is derived from and reflects society’s culture through fostering cooperation between the state and the public in orienting and comprehending the legal system (Naufal et al. 2014).

The principle of *ignorantia iuris neminem excusat* translates as ‘an individual’s ignorance of the law does not excuse’. This principle mandates that everyone is presumed to be aware of the law upon its publication in the official gazette or state news.

At this precise moment, the COVID-19 outbreak needs to be addressed. The law enforcement process has been ineffective in restricting the spread of COVID-19 because the present law is insufficient to provide legal clarity for the legal mechanism. Even though the regulation of positive laws for epidemic control in Indonesia began during the Dutch colonial period, the regulation of epidemics remains relatively easy, even when compared to the colonial period’s regulation (Rasjidi 1990:47).

Two types of legal construction arrangements continue to be a difficulty or legal concern in Indonesia’s disaster-management system (Hairi 2020:2–3):

1. Lack of a strong constitutional foundation in the face of a highly lethal and rapidly spreading epidemic such as COVID-19; for example, what if there is a disaster emergency for years until 2024, and presidential and regional elections cannot be held because of insufficient funding, and legislative elections must be postponed, who will hold power as the head of State if their term of office expires during the absence of elections? The Legislative Election and Presidential Election are scheduled for 21 February 2024, and the Regional Head Election or Pilkada is concurrently scheduled for 27 November 2024 (Syaiful 2021). Is it possible to tackle the problem using the PERPPU mechanism and the pressing urgency as a proxy for the level of danger? If PERPPU is used, what about the President’s power limits? Because the supreme legal principle is ‘*Salus populi suprema lex esto*’, which prioritises the public’s protection. In general, infectious disease eradication in Indonesia is accomplished by the following activities: early detection, patient identification, treatment, eradication of the illness’s source, immunity efforts (immunisation), and public education (Hasibuan 2020:28).
2. Statutory regulations originate as a result of the duality of legal groups and are relatively basic, whereas the resulting challenges range from state policy issues to criminal law enforcement.

Attempts at transformation of the UUPB are currently grappling with institutional challenges; funding, institutional and community participation, and measures to firmly enforce health norms have not been discussed (Ministry of Social Affairs RI 2020).

Djasmani (2011:366–368) argued in his book that according to some experts, the role of law as a tool for social engineering cannot operate in Indonesia since, from the Government’s perspective, legislation remains essentially a regulation or set of regulations enacted by a legislative body. Legislation is enacted to assist the government in carrying out its development mission. The formation of the Rule of Law from this vantage point is not based on the values of a plural society. The Rule of Law is created only from a deductive perspective (deductive logic based on the civil law legal system’s legacy). Law as a tool for social engineering will be successful if the Rule of Law is developed with consideration for the customary law that develops in society. The government must create space for the growth of customary law, integrate it into the national legal system, and make it the ideals upon which national law is based. Pound’s position contrasts with the School of History, which maintains that law grows and develops in lockstep with a habit-driven society (Djasmani 2011:366–368).

Rosco Pound emphasises the importance of the government carrying out two distinct features of government activity which are administrative aspects and legal aspects. This component assesses a government’s capacity to pick a broad hierarchy of individuals to do specific service jobs within the job and take the necessary actions to guarantee that the work is performed and to make corrections or modifications when circumstances change on a daily basis. This activity was critical in a primitive society and accounted for the majority of government action. Under contemporary society’s complicated and advanced conditions, this activity is insufficient unless stringent legal controls temper it.

Aspects of Legislation Formation: they must ensure legal certainty throughout the process of law enforcement. Indonesia already has an integrated legal framework for disaster management, including organisations that serve as the primary implementers, but not for coping with disease outbreaks, particularly in the aftermath of the COVID-19 epidemic. After 13 years of operation (2007–2020), UUPB discovered numerous issues, including ambiguous and overlapping legal definitions, authority to declare disasters, disaster level, lack of a formal law, looting, and various other issues.

**FIGURE 2:**
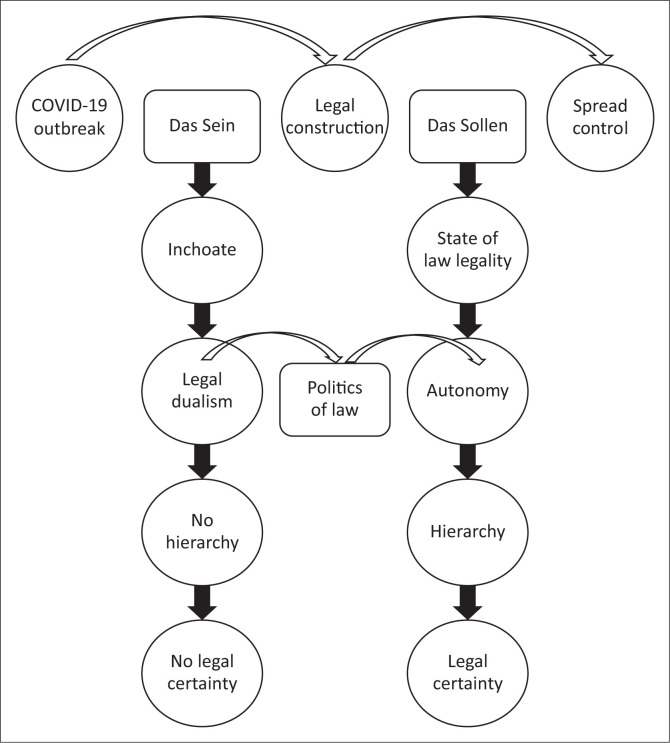
Indonesia’s legal framework for plague disaster management.

The UUPB is the primary legal framework governing Indonesia’s disaster-management system; additionally, all laws in the Republic of Indonesia, if any, that regulate disaster issues are disaster laws, including those governing diseases and the environment. Life and even fisheries laws are included in the disaster law system as they regulate in a state of force majeure. The Indonesian legal framework for p;agues disaster management can be seen in Figure 2.

In Indonesia, a law always requires implementing regulations, such as the UUPB, which requires six PPs and two Presidential Regulations, but the Presidential Decree on the mechanism for determining disaster emergencies has not yet been issued, despite the fact that decrees, particularly for regional heads, continue to be issued.

The general provisions of Law No. 6 of 2018 on quarantine state the following:

A public health emergency is a rare occurrence in which infectious illnesses and/or events are transmitted as a result of nuclear radiation, biological pollution, chemical contamination, bioterrorism, or food that constitutes a health threat and has the potential to spread across regions or countries.

In article 10, the Central Government establishes and terminates the ‘Public Health Emergency’. What is the difference between declaring a disaster and declaring an emergency? In point 10 of the UUPB’s general provisions, it is stated that:

A disaster emergency response is a series of activities carried out immediately following a disaster to address the disaster’s adverse effects, which may include the rescue and evacuation of victims and property, the provision of basic needs, the protection and management of refugees, and the rescue and restoration of infrastructure and facilities.

While emergencies differ in terms of the procedures that must be followed, the definition of what constitutes an emergency, and the breadth of action authorised by the responsible authority, each permit a flexible range of powers and measures necessary to contain a pandemic. Grogan (2020) lists four fundamental components of a constitutional provision authorising the creation of a state of emergency: (1) the criteria for its declaration; (2) transfer of authority; (3) limitations on their usage; and (4) measures for legislative or judicial oversight. For instance, the constitution may compel the Parliament to approve legislation authorising the declaration of a state of emergency either prior to or within a specified time period following the declaration (Molloy 2020:10).

The executive branch of power may declare a state of emergency in one of two ways. Firstly, a state of emergency is triggered by revolt or conflict. Secondly, the state of emergency triggered by a public health threat. Under such circumstances, David Davis observed, ‘the government has all the powers provided to it by the Constitution that are essential to maintain its existence’. This demonstrates that the bearer of government power, in the Indonesian context, the President, generally has the authority to exclude normal or ordinary laws through the enactment of emergency legislation. If it takes the form specified, then the President may take a range of actions, including declaring a state of emergency in the first context (Chandranegara 2021:45–70).

### Incorporation of disaster emergency law in the management of COVID-19

On 28 January 2020, the Head of the National Disaster Management Agency (BNPB) issued the Decree of the Head of BNPB RI Number 09 of 2020 concerning the Determination of the Status of Certain Disaster Emergency Situations in Indonesia because of COVID-19 (BNPB 2018:19). On 29 February 2020, the BNPB issued another Decree of the Head of the BNPB RI Number 13 Year 2020 regarding the Extension of the Status of Certain Emergency Disasters because of COVID-19 Outbreaks in Indonesia (BNPB 2020:1–2). This Decree of the Head of BNPB is based on Presidential Regulation Number 17 of 2018 concerning Disaster Management in Certain Circumstances, which states in consideration of letter b that:

That in certain circumstances where the status of a Disaster emergency has not been determined or has ended and/or has not been extended, but action is required or is still required to reduce disaster risk and its wider impact, it is necessary to assign assignments and authorities to BNPB to can carry out disaster management operations.

Article 1 defines Certain Circumstances as:

[*A*] situation in which the status of a Disaster Emergency has not been created or has ended and/or has not been prolonged, but action is required or continues to be required to mitigate the Disaster Risk and its broader consequences. (Article 1 point (1) of the Presidential Regulation 17 of 2018, page 3)

Firstly, The head of the BNPB’s choice is to ‘violate the law’, this is because the Head of BNPB’s decision is not a determination of national status because, according to the UUPB, the state alone has the authority to declare a state of emergency; in this case, the President, local governments, and the governors and regents or mayors. Secondly, while the Presidential Regulation does not regulate the determination of ‘certain status’, it does regulate the ‘handling of certain circumstances’ in which ‘emergency status’ has not been determined or has been determined but has expired. Thus, the Presidential Regulation does not control the BNPB’s jurisdiction to decide the state of a particular emergency but rather authorises the BNPB to continue or initiate its activity prior to determining whether or not the disaster emergency has been identified and established or ended.

Laws relating to disasters are laws in the sense that their contents are incorporated into heteronomous law, including the UUPB. The Government enacted Law No. 24 of 2007 on Disaster Preparedness and Management (UUPB) as a manifestation of Indonesia’s vulnerability as a disaster supermarket. The UUPB has also altered the paradigm of thinking about a disaster, which was initially viewed as an accident caused by an individual’s misfortune, then evolved into the responsibility and role of the Government alone, and has now evolved into the responsibility of a variety of parties, including the private sector and the community. This paradigm shift is motivated, in part, by an appreciation for the critical nature of multi-stakeholder disaster management.

With regards to disaster laws and policies, the UUPB mandates the issuance of six additional or implementing regulations, namely six Government Regulations (PP) and two Presidential Regulations (Perpres). According to Article 84 of the UUPB, the implementing regulations shall be issued no later than 6 months after the UUPB is promulgated.

Among a number of implementing regulations, the determination of disaster emergency status has not been issued. The Presidential Regulation establishing a disaster emergency status is critical because it establishes the legal certainty necessary for the legal structure for disaster management. The designation of a disaster emergency has ramifications for the expenditure of public funds, the use of public facilities, and deviations from regular rules, particularly in procurement. The disaster emergency designation has numerous legal implications, including a concept in which a decree can supersede the applicable law.

On 31 March 2020, the President also declared a Public Health Emergency in Indonesia in accordance with Presidential Decree No. 11 of 2020 Determination of a COVID-19 Public Health Emergency, which identifies COVID-19 as a disease capable of causing a Public Health Emergency and requires implementation in Indonesia. Additionally, on 14 April 2020, the President of the Republic of Indonesia declared the COVID-19 outbreak a national disaster through Presidential Decree No. 12 of 2020, which includes the decision to designate COVID-19 as a national disaster after considering the WHO’s decision to classify it as a pandemic and providing guidelines for regional heads to pay attention to national policies (BNPB 2016:15).

Both of these Presidential Decrees have their own legal basis, namely Presidential Decree No. 11 of 2020 concerning Public Health Emergency, which is based on Article 10 of the Undang-Undang Karantina Kesehatan or Law on Health Quarantine number 6 of 2018 (UUKK) and serves as the basis for implementing regional quarantine with the President determining which is based on the centralisation principle. Meanwhile, disaster emergency is defined to ensure that the Regional Head and President are not constrained, for example, in the procurement of goods under regular legislation and special legislative actions to ensure the seamless handling of disaster emergencies. The UUPB, which appoints regional and state heads based on the principle of regional autonomy, directs this disaster emergency.

### Towards an integrated outbreak emergency law

The distinctions of regulations must be reconciled as the starting point for developing a legal framework of disaster management in general and specifically for dealing with epidemics of the magnitude of COVID-19. Comparing different positive legal systems can contribute to the formation of a fundamental notion describing a new legal concept (Asshiddiqie & Safa’at 2006:11).

The Indonesian Government must enhance the Law on Infectious Disease Outbreaks to ensure that it meets all legal requirements, particularly in the event of a pandemic outbreak such as COVID-19, which has a profound effect on the nation, state, and society. The emergency law contained in the WPM Law should be developed in accordance with the legal certainty standards. Several drafts of the law in dispute include the following points.

### Outbreak emergency response

Outbreak Emergency Response is a terminology that crystalises many terminologies such as outbreak area, extraordinary event, public health emergency, among others. The legal idea proposed is Epidemic Emergency Status, a development of Disaster Emergency Status, which continues to apply, particularly in the case of non-epidemic calamities. Outbreak Emergency Response is a series of activities conducted immediately following the prevention and occurrence of outbreaks to address the outbreak’s adverse effects. These activities include assessment and research, outbreak prevention and management, medical treatment and quarantine, as well as economic and social impacts. Furthermore, epidemic emergency aid is a coordinated effort to address medical requirements, economic resilience, and social vulnerability in the event of an outbreak.

### Outbreak emergency status

Defining an epidemic when the status of an Outbreak Emergency is declared is critical to establishing a line of demarcation. In accordance with international law principles, numerous ease of access and laws will be implemented to expedite the process of epidemic containment. The government determines this status for a specified period of time based on the research conducted by the agency charged with public health.

### Plague management implementation

The implementation of epidemic control includes:

- rapid and precise epidemiological assessment of the major basic disease types to pinpoint the outbreak’s geographic location
- determination of the outbreak’s emergency status
- prevention
- medical treatment of those affected by the epidemic
- compliance with health protocols
- restrictions
- quarantine, territory quarantine, and lockdown
- satisfaction of fundamental regional quarantine and lockdown requirements
- immunisation
- economic management
- social impact management
- handling of the body
- other countermeasures.

### Appointment executor

The government determines the epidemic’s emergency level in accordance with the magnitude of the outbreak calamity. The President is responsible for declaring an emergency in the event of a pandemic outbreak; the governor is responsible for declaring an emergency at the provincial level; and the regent or mayor is responsible for declaring an emergency at the district or city level.

### Legal ease during outbreak emergency status

In the event of an epidemic emergency, the President and Regional Heads have easy access to mobilising human resources; deploying medical equipment; deploying logistics for the purposes of quarantine, regional quarantine, and locking; immigration control and licensing; procuring goods or services for medical, economic, and social needs; and managing and accounting for money and/or goods. Whoever intentionally obstructs the implementation of epidemic control must be identified, including violations of quarantine, regional quarantine, and locking, as well as the criminal sanctions system, which includes easy confinement and fines, but is based on legal certainty, benefit, and justice (Soeroso 2011).

## Conclusion

The Disaster Emergency Law in Indonesia has been initiated since the Dutch colonial time to state the country is in emergency. Disaster is one of the factors that contribute to the establishment of a state of emergency as self-contained, particularly since the law of UUPB 2007 was established. The concept of disaster emergency law in the Indonesian legal system is extremely diverse and widespread, adversely impacting the enforcement of COVID-19 emergency regulation. Emergency law has been used in the non-uniform principles, a violation of the principle of autonomy, a lack of legal certainty regarding the enforcement of health protocols, and a legal breakthrough in the disaster emergency concept system during the COVID-19 response.

This paper found that the Infectious Disease Outbreaks law (WPM Law 1984) has potentially to be utilised in dealing with the COVID-19 outbreak, particularly in the context of handling emergencies. However, currently this law is rarely employed in associating with legal regime of COVID-19 prevention.

It is advisable to revise the WPM Law to increase legal clarity, benefit, and justice and provide a punishment under the principles of the Rule of Law. The concept of epidemic emergency response must be self-contained and comprehensive in terms of objectives, legal certainty regarding actions and access, and the authority of heads of State and regions to declare epidemic emergencies in consistence with other emergency arrangements. Special quarantine preparations for epidemic emergencies must be properly controlled under the new WPM Law, involving the police and, in some cases, the military forces.
